# Sarcopenia parameters in active older adults – an eight-year longitudinal study

**DOI:** 10.1186/s12889-023-15734-4

**Published:** 2023-05-19

**Authors:** Kaja Teraž, Uros Marusic, Miloš Kalc, Boštjan Šimunič, Primož Pori, Bruno Grassi, Stefano Lazzer, Marco Vicenzo Narici, Mojca Gabrijelčič Blenkuš, Pietro Enrico di Prampero, Carlo Reggiani, Angelina Passaro, Gianni Biolo, Mladen Gasparini, Rado Pišot

**Affiliations:** 1 Institute for Kinesiology Research, Science and Research Centre Koper, Koper, Slovenia; 2 Faculty of Sport, University of Ljubljana, Ljubljana, Slovenia; 3 Department of Health Sciences, Alma Mater Europaea – ECM, Maribor, Slovenia; 4 Faculty of Medicine, University of Maribor, Maribor, Slovenia; 5 Department of Medicine, University of Udine, Udine, Italy; 6 Department of Biomedical Sciences, University of Padova, Padova, Italy; 7 National Institute of Public Health RS, Ljubljana, Slovenia; 8 Emeritus Professor of Physiology, University of Udine, Udine, Italy; 9 Department of Sport Science, Exelio SRL, Udine, Italy; 10 Department of Translational Medicine, University of Ferrara, Ferrara, Italy; 11 Medical Department, University Hospital of Ferrara Arcispedale Sant’Anna, Ferrara, Italy; 12 Department of Medicine, Surgery and Health Sciences, University of Trieste, Trieste, Italy; 13 Department of General Surgery, Izola General Hospital, Izola, Slovenia

**Keywords:** Elderly, Physical activity, Sedentary behavior, Longitudinal, Skeletal muscle disorder

## Abstract

**Backgroud:**

Sarcopenia is a common skeletal muscle syndrome that is common in older adults but can be mitigated by adequate and regular physical activity. The development and severity of sarcopenia is favored by several factors, the most influential of which are a sedentary lifestyle and physical inactivity. The aim of this observational longitudinal cohort study was to evaluate changes in sarcopenia parameters, based on the EWGSOP2 definition in a population of active older adults after eight years. It was hypothesized that selected active older adults would perform better on sarcopenia tests than the average population.

**Methods:**

The 52 active older adults (22 men and 30 women, mean age: 68.4 ± 5.6 years at the time of their first evaluation) participated in the study at two time points eight-years apart. Three sarcopenia parameters were assessed at both time points: Muscle strength (handgrip test), skeletal muscle mass index, and physical performance (gait speed), these parameters were used to diagnose sarcop0enia according to the EWGSOP2 definition. Additional motor tests were also performed at follow-up measurements to assess participants’ overall fitness. Participants self-reported physical activity and sedentary behavior using General Physical Activity Questionnaire at baseline and at follow-up measurements.

**Results:**

In the first measurements we did not detect signs of sarcopenia in any individual, but after 8 years, we detected signs of sarcopenia in 7 participants. After eight years, we detected decline in ; muscle strength (-10.2%; *p* < .001), muscle mass index (-5.4%; *p* < .001), and physical performance measured with gait speed (-28.6%; *p* < .001). Similarly, self-reported physical activity and sedentary behavior declined, too (-25.0%; *p* = .030 and − 48.5%; *p* < .001, respectively).

**Conclusions:**

Despite expected lower scores on tests of sarcopenia parameters due to age-related decline, participants performed better on motor tests than reported in similar studies. Nevertheless, the prevalence of sarcopenia was consistent with most of the published literature.

**Trial registration:**

The clinical trial protocol was registered on ClinicalTrials.gov, identifier: NCT04899531.

**Supplementary Information:**

The online version contains supplementary material available at 10.1186/s12889-023-15734-4.

## Background

Sarcopenia is a common skeletal muscle disorder [1] usually found in older adults and is defined as low muscle strength combined with low muscle quantity [1]. According to the different classifications of sarcopenia, the prevalence varies from 10 to 27% in individuals older than 60 years [2–4]. Moreover, the number of older adults with sarcopenia will increase tremendously in the next 30 years [5], which will have significant public health impact, e.g., increase in prevalence of physical disability, falls, injury-related falls, depression, hospitalizations, etc. There are several factors that contribute to the development and severity of sarcopenia: the ageing process itself [4, 6], inadequate nutrition [6, 7], physical inactivity - bed rest or sedentary lifestyle [3], chronic diseases [4, 6], drug treatments [4], and early life events [8]. Sarcopenia is highly associated also with adverse clinical outcomes such as falls, physical disability, fractures, cognitive impairment, hospitalisation, and consequently all-cause mortality [1, 9].

Among the most important factors that may contribute to a more rapid progression of sarcopenia are undoubtedly behavioral factors, such as physical activity (PA) and prolonged sitting time. According to Pišot, (2022) [10], our population has the most sedentary period in human history, but this is one of the factors (together with PA) that can be more easily influenced by society itself. A “sociology of sedentarism” is emerging to study this new phenomenon [11].

As reported by Gomes et al. (2017) [12], the prevalence of physical inactivity among older adults in Europe ranges from 5% in Sweden to 29% in Portugal [12]. Physical inactivity among older adults in Slovenia is 11.8% [12]. On the other hand, a systematic review by Steffl et al. (2017) [13] has shown the positive effect of PA on the alleviation of individual sarcopenia parameter (i.e., muscle strength, muscle mass and physical performance) and the occurrence of sarcopenia per se. The literature has already demonstrated that regular PA can reduce the incidence of sarcopenia [13–15].

PA for health or health enhanced physical activity (HEPA) is the amount of PA that is beneficial for health and can take place in leisure time, at work or in domestic duties [16]. Current recommendations for healthy older adults are: at least 150 min of moderate to vigorous aerobic PA per week to maintain functional abilities [17] and to achieve less than four sitting hours a day, in shorter periods [10]. In addition, muscle- and bone-strengthening activities that activate major muscle groups should be performed on regular level [18], preferable at least twice a week [17, 19].

Sarcopenia is assessed using several standardized tests to determine muscle strength, lean mass, and physical performance [1, 8]. Although one test from each category is sufficient to determine the presence of sarcopenia, many tests are validated by The European Working Group on Sarcopenia in Older Adults (EWGSOP). By using multiple tests, the condition of the human body can be addressed much more comprehensively. For example, it is known that muscle strength can be assessed with a handgrip strength test [1, 8, 20], but this test only measures upper limb muscle strength. Lower body strength may be better associated with functional activities and mobility tasks compared to handgrip strength, as it is required for daily activities such as transfers, walking, and climbing stairs. To comprehensively assess participants’ overall muscle strength, the Chair stand test and the 30-second chair stand test can be performed to evaluate lower limb muscle strength. Moreover, muscle mass can be assessed quantitatively or qualitatively [8]. Quantitative assessment of muscle mass can be conducted by various test such as bioelectric impedance, Dual-energy X-ray absorptiometry, magnetic resonance imaging etc. [1]. In addition, muscle mass can be calculated using predictive models that require information about the age, height, sex, body mass, and race of the individual to estimate it [21]. In this way, estimation of muscle mass facilitates longitudinal measurements where equipment failures or changes may occur due to the longer time window between measurements. Moreover, Narici et al. (2021) [22] proposed a new non-invasive ultrasound-based biomarker of the loss of muscle mass, called the ultrasound sarcopenic index. But the distinction between muscle quality and muscle quantity is an important aspect of functional assessment because two individuals with similar muscle mass are not necessarily capable of producing equal amounts of force. Similarly and counterintuitively, individuals with lower muscle mass can produce greater force than individuals with greater muscle mass [23]. An example of qualitative assessment of muscle mass is tensiomyography (TMG), which is increasingly used in research as a method to evaluate muscle quality [24–26]. In addition to quality assessment of muscle mass, TMG can be used also to monitor physical performance in older adults [27, 28]. Physical performance is defined as an objectively measured whole-body function related to locomotion, therefore again it should be measured comprehensively. Other tests to measure physical performance, recommended by EWGSOP [8] and EWGSOP2 [1], are gait speed, the Short Physical Performance Battery (SPPB) and the Timed-Up and Go test (TUG). Gait speed is widely used in practice [29], but TUG and the SPPB test are more complex tests as they include mobility, balance, and ability to perform activities of daily living [1, 30]. Along with gait speed and Short Physical Performance Batter (SPPB) we get a full insight of person’s physical performance.

To our knowledge, there are generally very few longitudinal studies [31–35] published on the prevalence of sarcopenia and PA in older adults. None of them were specifically interested in active older adults. Longitudinal follow-up of sarcopenia parameters in active elderly people can further identify the impact of regular physical activity and sedentary behaviour on all aspects of sarcopenia. From the cross-sectional studies is already well known that regular PA has beneficial role in development of sarcopenia [36, 37], but the exact impact of active lifestyles and thus active ageing is still unclear. Active older adults, who are currently underrepresented in sarcopenia research, can provide an ideal study cohort, as they tend to engage in regular physical activity. Therefore, we focused on the analysis of older adults who were regularly active (> 3000 MET/min per week) and examined any differences that may occur in active ageing over time with respect to sarcopenia parameters. The aim of this study is to examine the prevalence of sarcopenia parameters in active older adults over an eight-year period and to determine whether selected active older adults perform better on sarcopenia tests than the population average. We hypothesized that participants who reported more PA and less sedentary behavior (SB) in their lifestyle had lower decline in sarcopenia parameters after eight years. We were also interested in whether there were differences in sarcopenia parameters between participants who achieved HEPA levels through either work or planned PA and those who did not achieved HEPA level.

## Methods

### Participants

This is an observational longitudinal cohort study with 52 participants who participated in the baseline Physical Activity and Nutrition for Great Aging (PANGeA) mass measurement study [38] and then responded to follow-up measurements eight years later.

Goals of the PANGeA mass measurements were to conduct measurements to interpret the characteristics of healthy and active older adults. Among other cities, the baseline measurements were conducted also in city Koper (Slovenia), which included free-living older adults from the city of Koper and surrounding areas. and were performed in 2013. Eight years later participants from Koper and surrounding areas were invited for the follow-up measurements to examine the prevalence of sarcopenia parameters in active older adults over an eight-year period.

Inclusion criteria of baseline measurements were people between 60 and 80 years of age. Exclusion criteria of baseline measurements were unable to walk a distance of 2 km independently and continuously and/or severe cognitive decline, defined as MoCA score < 10 points (after correction for age and schooling). In addition, participants with acute illness or with a recent hospitalization (< 6 months), diabetics on insulin therapy, or on medications other than metformin were excluded from the baseline measurements. The exclusion criteria did not specifically target physical activity and sedentary behavior habits of participants.

**Fig. 1:**
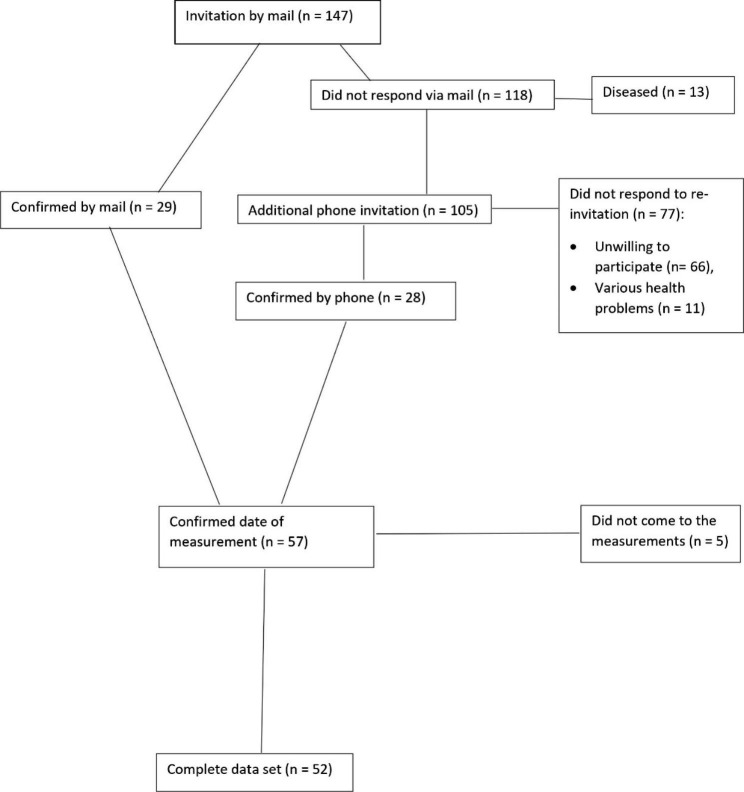
Flow diagram for the identification and screening of participants in follow-up measurements

Follow-up measurements were performed eight years later, in 2021. We invited all participants (147) from the baseline measurements in 2013 to participate in the follow-up measurements. Participants were invited to participate in the measurements by mail and also by telephone call. From the participants who were unwilling or unable to respond to the follow-up measurement, we obtain the reason for dropping out (death, unreachable, various health problems (unable to walk alone, hospitalization, presence of dementia). Between baseline (n = 147) and follow-up, 13 participants died, and 82 participants did not accept to participate in the follow-up measurements for various reasons (66 participants did not respond to the re-invitation, 11 participants could not participate because of health problems, and 5 participants made an appointment but did not come for the measurements), resulting in a final total analytic sample of 52 participants (response rate 35%). When comparing participants who participated in the first measurement only with participants who participated in both measurements, we found no differences in educational level, marital status, or self-rated quality of life. In addition, we found no differences in sarcopenia parameters and PA or SB categorization. Table 1 in the additional file 1 contains a more thorough comparison between participants who took part in both measurements and those who only participated in the baseline measurements. Results of older adults who participated in first and follow-up measurements were included and presented in this study. The process of collecting participants for follow-up measurements is shown in Fig. 1.

All participants signed an informed consent form. Before the study began, all participants completed a multidimensional questionnaire. Among other things, participants provided information on sociodemographic data, health status and drug therapy, and self-reported physical activity (c - GPAQ) [16].

### Study protocol

Participants visited the laboratory twice, once for baseline measurements (in 2013) and once for follow-up measurements (in 2021). To ensure the most accurate measurement results, both measurements (baseline and follow-up) were taken during the same part of the year. Data were collected in the laboratory. The measurements were conducted by specialist who had received training in performing specific assessment within their respective field. The measurements included anthropometric and body composition measurements, different motor tests (i.e., handgrip test, gait speed, SPPB, TMG) and the PANGeA Questionnaire which consisted of various questionnaires about sociodemographic data, general health status, general quality of life and drug therapy (descriptive variable). General quality of life was assessed through the Likert scale, from 1-poor to 5-excellent. Usual PA and SB was assessed by GPAQ [16], which is described in more detail in section “*Physical activity daily habits”*.

### Measurements

#### Anthropometric characteristics and body composition

Body mass (BM) was measured to the nearest 0.1 kg using a manual weighing scale (Seca 709, Hamburg, Germany) with the participant dressed only in light underwear and no shoes. Body height (BH) was measured to the nearest 0.5 cm on a standardized wall-mounted height board. Body mass index (BMI) was calculated as BM (kg) stature-2 (m). Appendicular skeletal muscle mass (ASM) was calculated based on the equation (Eq. 1) that was already used in several similar studies [3, 39, 40] and it is proposed by Lee et al., 2000 [21]. In Eq. 1, BM represents body mass (in kg), BH represents body heights (in m) and depending on the sex of the participant, we entered 0 for women and 1 for men.

1 $$ \begin{array}{c}ASM\,(kg)\, = \,0.244\,BM\, + \,7.8\,BH\,\\+ \,6.6\,sex\, - \,0.098\,age\, + \,0\, - \,3.3\end{array}$$

#### Sarcopenia parameters and physical daily habits

*Muscle mass*. Appendicular skeletal muscle mass (ASM) was estimated using the Eq. 1 proposed by Lee et al. 2000 [21]. Because muscle mass correlates with body size, the absolute level of muscle mass was adjusted for body size using body mass index (BMI) when quantifying muscle mass. ASM was then divided by BMI to obtain a skeletal muscle mass index (SMI). Based on previous recommended SMI cut-off points, a value of 0.789 for men and 0.512 for women was established [41, 42].

*Muscle strength.* Skeletal muscle strength upper extremities were assessed by the handgrip test. The handgrip test was evaluated with a hand dynamometer (Yamar, Patterson Medical, UK). The participant performed the test with dominant hand in a seating position with the elbow flexed at 90 degrees and positioned on the side, but not against, the trunk. The hand was positioned firmly on the dynamometer with the thumb pointing up. The average of three trails measured in kilograms was considered for further analysis. Weak handgrip strength was defined as < 27 kg for men and < 16 kg for women, using the average of the two handgrip measurements the dominant hand [1].

*Physical performance*. Physical performance was assessed by determining gait speed at a self-selected pace [1]. Participants walked from side to side for one minute and thirty seconds on a 4-metre path that was on a flat and non-slippery surface. On each side of the 4-metre track, participants had an additional 1-metre track with cones on which they turned around. Only the stale, self-selected gait (without turns and decelerations/accelerations) was observed and averaged as the final result. Slow gait speed was defined using EWGSOP2 [8] reference values of < 0.8 m/s.

*Physical activity daily habits*. PA was assessed using a validated self-assessment questionnaire, the Global Physical Activity Questionnaire (GPAQ) [16], consisting of 16 questions divided into three domains and a question on SB. The three domains are as follows: Activity at Work, Travel to and from Places, and recreational activities. Since the study mainly involved retired elderly people, we included all activities that elderly people have to do at home or around the house (e.g., gardening, working in the vineyards, olive groves, etc.) in the scope of occupational activities. The GPAQ analysis uses metabolic equivalents (METs) and assigns a total of 4 METs and 8 METs for moderate and vigorous activity, respectively, per time spent in the specific activity. Each questionnaire was reviewed by a researcher in collaboration with the respondent and corrected as indicated in the GPAQ guidelines. WHO recommendations specify a threshold for total PA MET -minutes-week-1 of 600 that must be achieved to be considered healthfully active.

SB was assessed using the Global Physical Activity Questionnaire with a question, “On a typical day, how much time do you spend sitting or lying down?“ The person answered in hours and minutes.

### Measurements assessed at follow-up

At the follow-up measurements, we performed additional sarcopenic motor tests to assess the sarcopenia parameters of our sample in more detail.

*Lower extremity muscle* strength was measured using both the Chair stand test and the 30s chair stand test (30CST). Both the Chair stand test and the 30CST were performed in this study in accordance with established protocols [43, 44]. Participants were instructed to stand up from a seated position as quickly as possible. Both tests were performed at the same time, and after 5 ascents, the administrator noted the time required. At the same time, the maximum number of attempts to stand up in 30 s was determined. The Chair stand test measures the time in seconds required to complete five repeated stand-ups. The 30CTS measures the maximum number of chair holds completed in the 30 s of the test. Performance impairment on the Chair stand test was defined using the EWGSOP2 [1] cut off > 15 s. We also compare the results of our sample with previously published studies with similar characteristics. Reference scores for the Chair stand test were 11.2 for men and 12.2 for women and for the 30CST were 13.8 for men and 13.7 for women [45].

*Tensiomiography*. Skeletal muscle mechanical contractile properties were assessed using a tensiomiography (TMG) device (TMG-ZD1, TMG-BMC d.o.o., Slovenia) in three muscles of the right leg: vastus lateralis (VL), bices femoris (BF), and gastrocnemius medialis (GM). Participants were asked to relax on a bed supine (VL), with their knee angle fixed at 30 degrees of flexion, or prone (BF, GM), with the knee angle flexed at 5 degrees and the ankle in a neutral position. Foam pads were used to support the joints. The measuring point for TMG assessment was the thickness part of each muscle belly, as described previously [46, 47]. Briefly, two self-adhesive electrodes (PALS, Axelgaard) were positioned 5 cm distal (cathode) and 5 cm proximal (anode) to the thickest part of the muscle belly. No skin preparation was needed. Muscle contraction was triggered with a single maximal rectangular electrical stimulus of 1ms duration and maximal amplitude. The linear displacement sensor detected the transverse radial enlargement as a response in time domain. Two contractile parameters were estimated: Dm as a maximal amplitude of the response and Tc as the time between 10% and 90% of Dm. In each muscle, two maximal TMG responses were recorded, and an average of estimated parameters were taken for analysis [48, 49].

*The SPPB* is an assessment which includes 5 tests for lower limb function including balance, strength and mobility. It was calculated based on EWGSOP definition [8]. Three individual measures of physical performance including gait speed, balance test and Chair stand test. The aim of the balance tests was to stand for 10 s with the feet together in side-by-side, semi-tandem and tandem positions and unaided, with the test progressing in difficulty after successful completion. Gait speed was assessed as previously described (see “gait speed), the Chair stand test was performed as described above (see “Chair stand test”). The total SPPB score ranging from 0 to 12; higher scores indicate better performance [50].

*TUG* is a valid measure of mobility, balance, and the ability to perform activities of daily living. Participants were seated in an arm chair and were timed on their ability to stand up from the chair, walk a 3 m course, turn around, walk back to the chair and sit down again (in seconds) [51].

*Sarcopenia*. To categorize the severity of sarcopenia, participants were divided into three groups according to EWGSOP2 [1].

- Probable sarcopenia was confirmed if low muscle strength was identified (handgrip strength lower than 27 kg for men and 16 kg for women and/or chair stand test was completed in more than 15 s for five rises.
- Sarcopenia was confirmed if low muscle strength was noted in addition to SMI lower than 0.789 for men and 0.512 for women was identified.
- Severe sarcopenia was confirmed when participants were diagnosed with low muscle strength and low physical performance (gait speed was defined as slow when it was lower than 0.8 m/s).

### Participant categorization

For purpose of this study, participants were divided into groups according to two different ways, according to their level of PA or their level of sedentary behaviour.

Their level of PA was categorized depending on the achievement of the recommended HEPA level. The cut point for sufficient total activity was 3,000 MET minutes per week accumulated over 7 days. We divided participants into those who achieved HEPA level mostly through planned PA (HEPAP), those who achieved HEPA level mostly by working (HEPAW) and those who did not achieve the HEPA level (HEPAN).

Moreover, we divided participants by their level of sedentary behaviour: those who reported sitting four hours or less per day (SB-L) and those who reported sitting more than four hours per day (SB-H).

### Statistical analysis

All anthropometric characteristics and sarcopenia parameters are described with mean and standard deviation (SD). Normal distribution was confirmed by visual inspection using histogram and Q-Q plots and analytically by Kolmogorov-Smirnov test. To examine the prevalence of sarcopenia parameters in active older adults over eight years we used the following statistical methods; general characteristics and sarcopenia parameters of the sample from baseline and follow-up measurements were assessed using paired sample t-test. The correlation between PA, sedentary behaviour, and each sarcopenia parameter (handgrip strength, SMI, and gait speed) was examined using Pearson’s correlation coefficient. First, the relationship between PA and SB and handgrip strength, skeletal muscle index, and gait speed at baseline was examined. Second, the association between follow-up PA and SB and loss of handgrip strength, skeletal muscle index, and gait speed over an 8-year period was examined. To determine whether selected active older adults perform better on sarcopenia tests than the population on average, the results of secondary measures of sarcopenia parameters were used. Results of secondary measures of sarcopenia parameters (Chair stand test, TUG, SPPB, and TMG) were compared using a one-sample t-test with data from different populations/studies [45, 52–54] that included Caucasian older adults aged between 60 and 67 years, without any specific diseases stated. TMG values in sarcopenic and non-sarcopenic participants were assessed using nonparametric The Mann-Whitney U-test. Correlations between TMG values and selected sarcopenia parameters were examined using Spearman’s rank correlation coefficient. To compare different types of achieved HEPA and SB with sarcopenia parameters, we used mixed linear models, to account for between-subject variability (differences between groups either HEPA or SB categories at baseline) and within-subject variability (differences between baseline and follow-up measurements), adjusted for age. HEPA or SB and time were used as fixed effects and age was used as random effects. Bonferroni post hoc corrections were applied to determine differences between the groups. Before follow-up measurements, handgrip strength was defined as the primary outcome variable for sample size and power analysis. G*Power [55] was used for sample size and effect size calculation, using Cohen’s d (α = 0.05, power = 0.95). The literature [56] estimates that the decrease in handgrip strength after eight years will be 5.2 kg in men and 2.9 kg in women. With a chosen alpha of 0.05 and power of 0.95, a sample size of 30 men (effect size 0.625) and 39 women (effect size 0.538) was required.

All statistical analyses were performed using IBM SPSS Statistics 22 (SAS Institute, Cary, NC, USA).

### Results

The characteristics of the older adults who participated at baseline and at follow-up are described in Table 1. The mean age of included participants at baseline was 68.3 ± 5.4 years (57.7% women). The prevalence of sarcopenia at baseline was low, with zero participants reporting sarcopenia criteria according to EWGSOP2 [8]. After 8 years, we detected 13% prevalence of sarcopenia: four participants were found to have probable sarcopenia (low skeletal muscle strength), two participants met sarcopenia criteria (low muscle strength and low muscle quantity or low muscle quality), and one participant developed severe sarcopenia (low muscle strength, low muscle quantity or quality, and low physical performance) [1].

**Table 1:** Characteristics of the pooled study sample at a baseline and follow-up

	Baseline	Eight-years follow-up	*p-*value
Age (year)	68.3 ± 5.4	75.9 ± 5.3	< 0.001
Women, *n* (%)	30 (57.7)		
**Education**, ***n*(%)**			
Primary	0		
Secondary	29 (55.8)		
College/University	23 (44.2)		
**Marital status** ***n*** **(%)**			
Married	28 (53.8)	27 (51.9)	
Other	24 (46.2)	25 (48.1)	
Number of comorbidities/people	3.4 ± 2.3	3.5 ± 2.1	0.121
Number of medications/people	1.9 ± 1.7	2.9 ± 2.4	0.001
Quality of life	2.2 ± 0.6	4.0 ± 0.5	< 0.001
Body height (cm)	167.8 ± 9.1	166.2 ± 8.9	0.007
Body mass (kg)	73.3 ± 12.9	73.0 ± 14.1	0.346
Body mass index (kg/m^2^)	24.9 ± 6.3	26.3 ± 4.0	0.117
Handgrip strength (kg)	35.4 ± 14.9	31.8 ± 12.2	< 0.001
Skeletal muscle index	0.92 ± 0.21	0.87 ± 0.22	< 0.001
Gait speed (m/s)	1.4 ± 0.2	1.0 ± 0.2	< 0.001
**Sarcopenia**, ***n***			
Probable sarcopenia	0	4	
Sarcopenia	0	2	
Severe sarcopenia	0	1	
Physical activity (METmin/week)	5170 ± 3112	3876 ± 2665	0.030
Sedentary behaviour (min/day)	260 ± 115	134 ± 75	< 0.001

*SMI – skeletal muscle index

All selected sarcopenia parameters were different between baseline and follow-up; handgrip strength (t = 7.963, *p* < .001; Cohen’s d = 0.52), SMI (t = 6.915; *p* < .001; Cohen’s d = 1.67) and gait speed (t = 12.725; *p* < .001, Cohen’s d = 0.22) (Table 1). Furthermore, there was also difference in self-reported amount of PA (t = 2.243; *p* = .030; Cohen’s d = 0.44) daily sedentary behaviour (t = 6.6561, *p* < .001; Cohen’s d = 1.38) and the number of prescribed medication (t = -3.604; *p* = .001; Cohen’s d = 0.52) (Table 1).

After performing additional statistical analyses with mixed linear models we confirmed difference in time for handgrip strength (*p* < .001), SMI (*p* = .004) and gait speed (*p* < .001) in HEPA (Table 2) and for handgrip strength (*p* < .001) and gait speed (p < .001) in SB (Table 3) after adjusting for age (Table 3). We did not find any differences between groups (HEPA and SB) with exception of SMI (*p* = .030) for HEPA groups; post-hoc analysis showed differences between HEPAW and HEPAN groups (*p* = .031) in SMI.

**Table 2:** Main results of mixed linear models and sarcopenia parameters at baseline and 8-years follow-up measurements according to the type of physical activity, adjusted for age

	Baseline			Eight years follow-up					
	HEPAW	HEPAP	HEPAN	HEPAW	HEPAP	HEPAN	P_TIME_	P_HEPA_	P_TIMExHEPA_
Handgrip strength (kg)	37.9 [33.5, 42.2]	39.1 [33.3, 44.8]	38.2 [34.3, 42.2]	31.6 [27.6, 35.6]	33.3 [28.6, 37.9]	32.3 [27.8, 36.7]	< 0.001	0.729	0.987
SMI	0.98 [0.90, 1.1]	0.89 [0.79, 1.0]	0.88 [0.81, 0.95]	0.88 [0.81, 0.96]	0.87 [0.79, 0.96]	0.82 [0.73, 0.90]	0.004	0.030	0.570
Gait speed (m/s)	1.4 [1.3, 1.5]	1.3 [1.1, 1.5]	1.4 [1.3, 1.5]	0.97 [0.88, 1.06]	1.02 [0.90, 1.14]	1.15 [1.04, 1.2]	< 0.001	0.150	0.113

*SMI – skeletal muscle index, HEPAW - achieved HEPA level mostly by working; HEPAP - achieved HEPA level mostly through planned physical activity; HEPAN – did not achieved HEPA level

**Table 3:** Main results of mixed linear models and sarcopenia parameters at baseline and 8-years follow-up measurements according to the type of SB, adjusted for age

	Baseline		Eight years follow-up				
	SB-L	SB-H	SB-L	SB-H	P_time_	p_SB_	P_TIME x SB_
Handgrip strength (kg)	38.2 [34.7, 41.7]	35.4 [29.0, 41.9]	32.1 [28.5, 35.7]	32.7 [28.80, 36.7]	0.005	0.535	0.306
SMI	0.93 [0.86, 0.99]	0.83 [0.71, 0.96]	0.86 [0.79, 0.93]	0.86 [0.78, 0.93]	0.465	0.174	0.171
Gait speed (m/s)	1.4 [1.3, 1.4]	1.5 [1.3, 1.7]	1.0[0.93, 1.1]	1.1 [1.0, 1.2]	< 0.001	0.195	0.997

*SMI – skeletal muscle index, SB-L – low sedentary behaviour, SB-H – high sedentary behaviour

We found positive correlation between self-reported PA at baseline and measured muscle strength (r = .392, *p* = .006, r^2^ = 15.4%) with handgrip strength test.

At follow up measurements we did not found any correlation between self-reported PA or SB and selected sarcopenia parameters.

Table 4 shows secondary measurements of sarcopenia parameters compared with data from other populations or studies that included healthy older adults. Compared with reference values where individuals were older adults without abnormalities such as use of assistive devices, multiple falls and were well enough to undergo the fitness assessment, men performed better on tests assessing physical performance (TUG (t = -5.296; *p* < .001; Cohen’s *d* = -1.15) and SPPB (t = 6.253; *p* < .001; Cohen’s *d* = 1.38)) while females were better in Chair stand test (t = -3.046; *p* = .005; Cohen’s *d* = − 0.58), TUG (t = -8.483; *p* < .001; Cohen’s *d* = -1.57), and SPPB (t = 5.125; *p* < .001; Cohen’s *d* = 0.91).

**Table 4:** Secondary outcomes of sarcopenia parameters in follow-up measurements in comparison with data from other population

	Men (N = 22)			Women (N = 30)		
	Mean ± SD	Reference value	*p* value	Mean ± SD	Reference value	*p* value
Chair stand test (sec)	10.1 ± 2.9	11.2(45)	0.096	10.3 ± 3.3	12.2(45)	0.005
30s chair stand test (rep)	15.6 ± 5.4	13.8(45)	0.136	13.8 ± 4.9	13.7(45)	0.883
Timed up and go test (sec)	6.9 ± 2.0	9.2(52)	< 0.001	7.0 ± 1.4	9.2(52)	< 0.001
SPPB (point score)	11.3 ± 0.8	10.2(53,57)	< 0.001	11.2 ± 1.1	10.2(53)	< 0.001

Statistically significant at p < .05

SPPB – short physical performance battery

In addition, we found different values in TMG measurements (assessed at follow-up) between our population and Reference value (Table 5). Biceps femoris and Vastus Lateralis had lower Dm in our population in comparison to the Reference value [54]. To support this, we also found lower DmVL in older adults with sarcopenia parameters when compared to older adults without sarcopenia parameters (Z = 2.097, *p* = .036).

**Table 5:** Tensiomyography data in comparison to data from other populations

	Mean ± SD	Reference value [54]	*p* value
Bices femoris Tc (ms)	41.3 ± 19.1	42.0	0.796
Bices femoris Dm (mm)	4.3 ± 2.8	5.9	< 0.001
Gastrocnemius medialis Tc (ms)	27.7 ± 9.5	29.9	0.106
Gastrocnemius medialis Dm (mm)	3.7 ± 1.9	3.9	0.467
Vastus lateralis Tc (ms)	25.1 ± 5.1	25.0	0.543
Vastus lateralis Dm (mm)	4.2 ± 1.9	5.4	< 0.001

Statistically significant at *p* < .05

Tc – contraction time, Dm – maximal displacement

Furthermore, there was a positive moderate correlation between participant’s sarcopenia parameters such as SMI, handgrip strength, gait speed and DmVL (rho = 0.531, *p* < .001; rho = 0.405, *p* = .004; rho = 0.341, *p* = .015, respectively) and negative correlation between Chair stand test, TUG and DmVL (rho = 0.359, *p* = .011; rho = 0.370, *p* = .008, respectively). Moreover, we found negative correlation between TUG and DmGM (rho = − 0.315, *p* = .028).

## Discussion

The purpose of this longitudinal study was to investigate the change in sarcopenia prevalence over an 8-year follow-up period in initially active, non-sarcopenic, older adults (68.4 years old at baseline) and to determine how sarcopenia parameters changed and how they correlated with this change. Although the response rate was low (35%), it is in consisted with previously reported results [35, 57] and provides important information about the sarcopenia parameters in active older adults. No sarcopenic individuals were identified in the initial selected sample of active older people, but we found a prevalence of 13.5% sarcopenia after 8 years. The results are consistent with the reported prevalence of sarcopenia in the European population aged 60–80 years [3, 4, 58]. There was a difference between baseline and follow-up measurements for all selected sarcopenia parameters; handgrip strength, SMI, and gait speed were lower in the follow-up measurements for participants who took part in both measurements. This results were expected because of the well-known decline in muscle strength, muscle mass, and physical performance with progressive age [31, 59]. Nevertheless, the mean values of most sarcopenic traits in our participants are still above the limits set by EWGSOP [8] and EWGSOP2 [1].

On average, participants experienced 0.8% decline in muscle mass and 2.5% decline in muscle strength per year. This is in line with existing literature reporting muscle mass decreases at a rate of 0.5-1% and muscle strength declines at a rate of 2–3% per year after the age of 50 [59]. Maintaining functional fitness is necessary to prevent disability in an ageing population [45], therefore, the right amount and type of PA is essential for maintaining muscle strength and muscle mass. Our participants reported being less physically active after eight years, but in general still meeting health-enhancing physical activity (HEPA) standards [16]. Nevertheless, there was no difference in sarcopenia parameters among participants who achieved HEPA by work or by planned PA. On the other hand, we found differences in SMI among participants who achieved HEPA by work in comparison to participants who did not achieved HEPA. In addition, SB of participants who took part in both measurements was lower after eight years. This finding is encouraging, because it is known that the increasing time spent in SB is associated with increased prevalence in sarcopenia and sarcopenic obesity in older adults [3, 60]. Smith et al. (2020) [3] concluded that a sedentary lifestyle of more than 11 h per day is associated with sarcopenia. Between baseline and follow-up measurements, the COVID-19 pandemic occurred, which has generally resulted in less active and more sedentary population [61]. Despite the fact that the COVID − 19 pandemic, which has a major impact on the lives of all people, Pišot et al. (2020) [62] did not find a decrease in PA and an increase in SB among Slovenian living in the coastal zone. Nevertheless, the finding that SB is lower after eight years should be interpreted with caution, as the literature reports that GPAQ results can often underestimate or overestimate the actual value of SB or PA [63, 64].

To gain better insight into the physical characteristics of our participants, we performed additional tests to assess sarcopenia parameters. It is known that muscle strength can be assessed with a handgrip test [1, 8, 20], but this test only measures upper limb muscle strength. To access lower limb strength, we used Chair stand test and 30CTS test. Overall, both men and women performed better on both tests compared with reference values [45] (only women in Chair stand test).

For a more detailed look at physical performance, we used TUG and the SPPB test for follow-up measurements. With better TUG and SPPB values of our sample in the comparison to the population [52, 53, 65], we can only further confirm the very good physical fitness. In addition, the results of our follow-up measurements confirm that participants have better physical performance than individuals representing older adults with an average active lifestyle. Although the data on the prevalence of sarcopenia in our sample are consistent with the published literature, after eight years our participants still had better outcomes than those published in the literature that studied community dwelling older adults of similar age to our sample. One possible explanation for this is regular and varied PA. Our participants reported a variety of physical activities that they regularly include in their daily routine, such as walking, planed PA such as yoga, stretching, balance exercises, gardening, working in the vineyards, olive groves etc. Relatively small increases (> 48 min more) in various PA over already recommended doses can have significant effects on gait speed [66], which is the most important indicator of physical performance in older adults [1].

To better assess the muscles and thus get a better insight into the muscle quality, TMG measurements, performed during the follow-up measurements. We already know that there is an age-related slowing of all muscles over time [46]. Prolonged muscle Tc in the elderly may result in lower performance outcomes in functions of daily living [46]. Comparing our results with selected literature [54] data of similar age groups, there was no difference between the Tc of all observed muscles. On the other hand, we found lower Dm in VL and BF in our participants when compared to reference values [54]. It has already been established that the increase in Dm measurements can provide evidence of preatrophic changes [49], so we would assume that participants who developed sarcopenia parameters will show increased values in Dm. On the contrary, participants with sarcopenia had decreased values of Dm in comparison to participants without sarcopenia. This is a novel finding and is similar for all selected muscles, but because of the small sample, we could statistically confirm it only for the VL. Possible explanations for the phenomenon can suggest that sarcopenic individuals can have lower contractility of the muscle with lower amount of contractile elements in the muscle, higher fat and connective tissue infiltration, a reduced number of sarcomeres, smaller pennation angle, thinner muscle tissue [67]; possible causes that have already proved a link for decreased Dm. The fact that we found no differences in the Tc parameter, a parameter previously linked to the proportion of myosin heavy chain proportion [48], between sarcopenic and nonsarcopenic participants supports equal proportion of muscle fibre phenotypes between both groups. And indeed, sarcopenic ageing causes the loss of muscle fibres, the proportion of type I fibres to increase; however, a definitive shift from fast to slow is not empirically supported [68]. Further, we confirmed correlation between all sarcopenia measures (positive: SMI, handgrip strength, gait speed; and negative: TUG, Chair Stand) with DmVL. These data suggest that participants who performed worse on these tests had lower DmVL Interestingly, also DmBF (but not DmGM) was lower in study participants when compared to reference values [54], however, we could not confirm lower DmBF in sarcopenic than in non-sarcopenic, due to low sample size. On the other hand, muscle Tc values were comparable to the reference values the phenomenon is certainly interesting and would be worth investigating in more detail.

Although our participants performed better than the average age group on most tests, the decline in all functions measured longitudinally was still comparable to that expected [4, 59, 69]. Therefore, this does not necessarily mean that self-selected PA or an active lifestyle is sufficient to combat sarcopenia. However, we can conclude that despite the age-related decline in measured functions, the elderly entered old age in better condition and, therefore, this decline had less negative impact on the factors already demonstrated. This finding is not to be neglected, because in the literature it was already stated many times [40, 70, 71], that healthy ageing has a positive effect on the physical and mental functions. As in a study conducted with the same sample, we already concluded that older people with an active lifestyle reported higher ratings of quality of life, general health, and overall well-being [72]. All listed positive effects of physical activity thru life-course and in later life are special focus of the key strategic documents like WHO Decade of Healthy Ageing [73], which states that all older adults, irrespective of the level of intrinsic capacity, should have opportunities to optimize functional ability in order to enjoy what they value most. Slovene Longevity strategy [74] is based also in PANGeA results [75] and encourages increased physical activity, along with healthy nutrition for older ages, to prevent sarcopenia in rapidly ageing population. Additional focus in Strategy is dedicated to older people from lower socioeconomic strata, specifically planning for physical activity programs for that subpopulation group.

Regular and sufficient PA in combination with low SB has been repeatedly shown to have a significant effect on the deterioration of the development of sarcopenia parameters [3, 13–15, 60, 76]. Varied PA also has an important positive impact on strength, mobility, balance, ability to perform daily activities, etc. [77–79]. Long-term follow-up of older performing various regular PA has shown positive results in favor of an active lifestyle.

### Limitations to the study

Some limitations should be addressed. The prevalence of sarcopenia determined in this study is comparable to other studies using the EWGSOP2 definition. However, a recent studies [2, 80] comparing different definitions of sarcopenia highlighted the limited sensitivity of the EWGSOP2 definition for the diagnosis of sarcopenia, therefore the “true” prevalence is difficult to determine. Moreover, prevalence of sarcopenia was likely higher than in the analysis sample because sarcopenic participants were more likely to be classified as lost-to-follow-up. Secondly, PA was determined by self-report. This resulted in overestimation of PA levels and underestimation of SB. Objective measurement of PA could improve the reliability of PA data. However, we believe that self-report gives a good indication of whether a participant is not active at all or is very active. Third, muscle mass was calculated utilizing a population equation and not direct assessment. In this sense, this equation did not consider some common anthropometric measures, such as hip circumference and the proposed equation was validated only in the USA, whilst no direct evaluation was made in Europe. Nevertheless, given the widespread use of the equation in previously published studies, we believe that its use is justified. Fourth, this study did not control for factors such as the occurrence of diseases, changes in nutrition, changes in residency, and life events that could have altered the outcome over the eight-year period. Fifth, no midterm assessment of PA, SB, socioeconomic status, or sarcopenia parameters was performed during the 8-year follow-up period to provide a more detailed insight into the nature of the variation in observed parameters. Finally, our study may have been underpowered to detect realistic changes over an eight-year period; a larger sample may be needed to detect longitudinal changes in active older adults.

## Conclusions

Over the eight years, there was an expected deterioration in both motor skills and physical characteristics of the participants. However, our participants performed better on most tests in comparison to older adults who are not categorized as active older adults. Their self-reported levels of PA and SB were higher and lower, respectively, than the recommended levels. TMG data revealed no change in muscle fiber type composition but lower muscle contractility in the whole sample as well as in sarcopenic when compared to non-sarcopenic. Therefore, TMG could be a good candidate for muscle quality assessment for the sarcopenia classification. More studies could provide sufficient data to validate it as a relevant indicator to be included among standard sets of measurements.

In conclusion, although our sample showed a smaller decrease in sarcopenia parameters compared with a reference sample, future studies need to investigate whether an active lifestyle can indeed prevent deterioration of motor skills and mental health. To this end, an appropriate research design must be used.

## Electronic supplementary material

Below is the link to the electronic supplementary material.

Supplementary Material 1

## Data Availability

The datasets used and/or analysed during the current study are available from the corresponding author on reasonable request.

## Abbreviations

Dm: Maximal displacement
EWGSOP: European Working Group on Sarcopenia in Older Adults
GPAQ: Global Physical Activity Questionnaire
HEPA: Health enhanced physical activity
HEPAN: Not acheived health enhanced physical activity
HEPAP: Health enhanced physical activity mostly through planned physical activity
HEPAW: Health enhanced physical activity mostly by working
MET: Metabolic equivalents
PA: Physical activity
PANGeA: Physical Activity and Nutrition for Great Aging
SB: Sedentary behavior
SB-L: Low sedentary behaviour
SB-H: High sedentary behaviour
SMI: Skeletal muscle index
SPPB: Short physical performance battery
Tc: Contraction time
TUG: Timed Up and Go test
