# Supplementary material for: Sarcopenia parameters in active older adults – an eight-year longitudinal study
Source: BMC Public Health. 2023 May 19;23:917. doi: 10.1186/s12889-023-15734-4 (PMC10197056; doi:10.1186/s12889-023-15734-4)
Supplement: Supplementary file 1 — Supplementary Material 1 [file 12889_2023_15734_MOESM1_ESM.pdf]

Table 1

Comparison of baseline characteristics in responders to follow-up measurements and non-responders

	Non-responders	Responders	t-test (p value)
Age	68.5 ± 5.8	68.3 ± 5.4	.173 (.863)
Body mass (kg)	76.2 ± 13.9	73.3 ± 12.9	1.199 (.233)
Body height (cm)	165.5 ± 8.7	167.8 ± 9.1	-1.506 (.134)
Body mass index (kg/m^2)	27.6 ± 3.7	24.8 ± 6.4	3.351 (.001)
Handgrip strength (kg)	35.4 ± 9.8	35.4 ± 14.9	-1.560 (.121)
Appendicular skeletal muslce mass (kg)	23.7 ± 6.3	23.2 ± 6.4	.451 (.653)
Gait speed (m/s)	1.3 ± 0.2	1.4 ± 0.2	-1.437 (.153)
Physical activity (METmin/week)	4713 ± 4733	5170 ± 3112	-.307 (.759)
Sedentary behavior (min/day)	282 ± 188	260 ± 115	.508 (612)
